# Supplementary figures and images for: Circular RNA HMGCS1 sponges MIR4521 to aggravate type 2 diabetes-induced vascular endothelial dysfunction
Source: eLife. 2024 Sep 5;13:RP97267. doi: 10.7554/eLife.97267 (PMC11377038; doi:10.7554/eLife.97267)

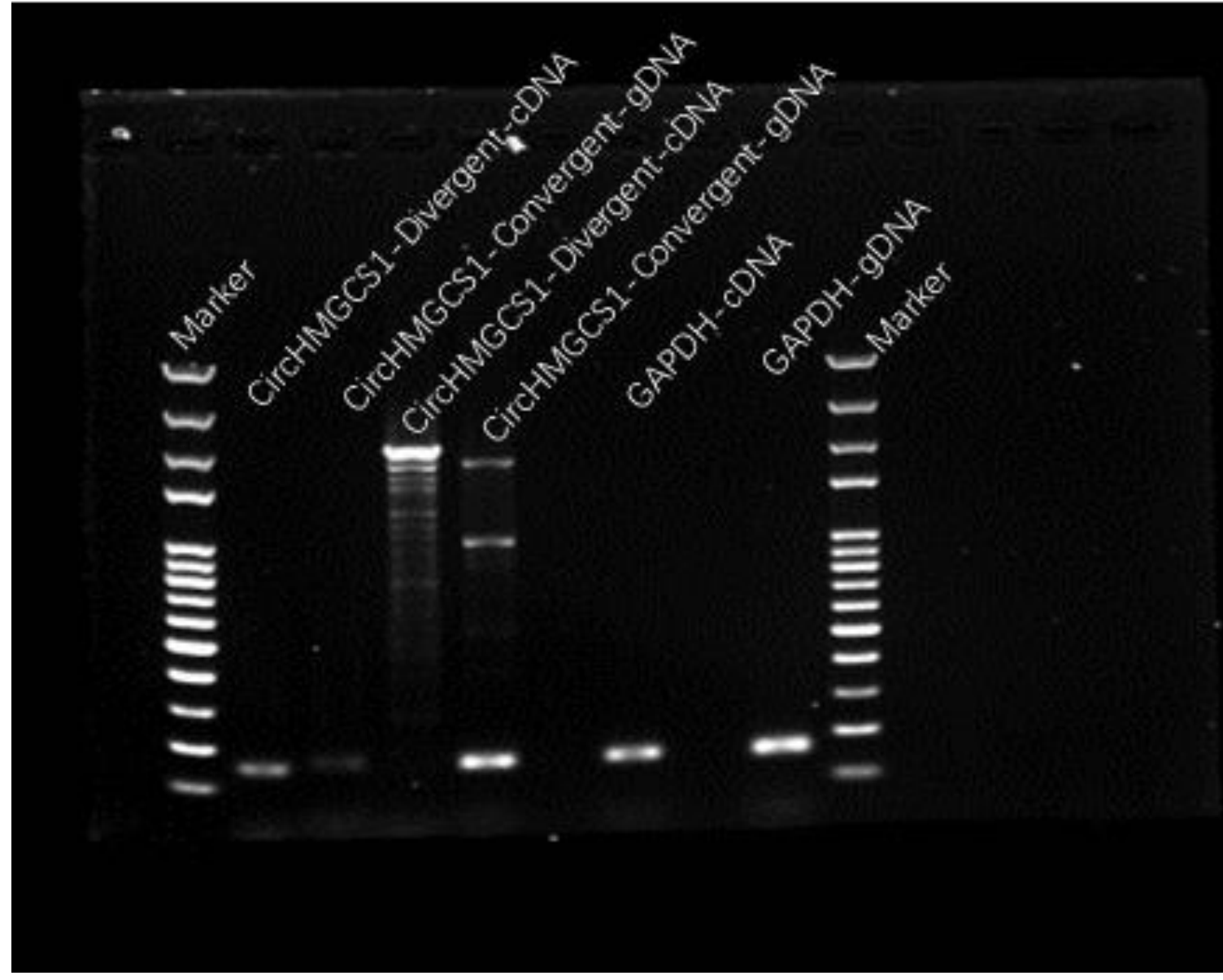

Supplement: Figure 1—source data 1. [file elife-97267-fig1-data1.zip › Figure 1-source data 1/Figure 1-Source data 1 Uncropped and labeled gels for Figure 1.pdf]

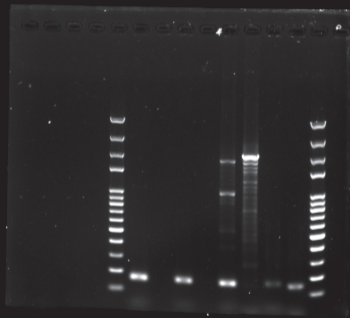

Supplement: Figure 1—source data 2. [file elife-97267-fig1-data2.zip › Figure 1-source data 2/Figure 1-source data 1 Raw unedited gels for Figure 1.pdf]

ET-1

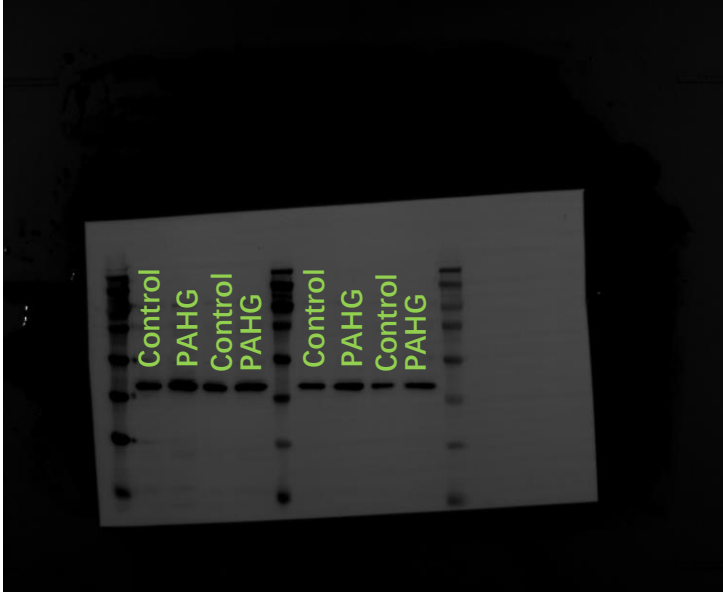

ICAM1

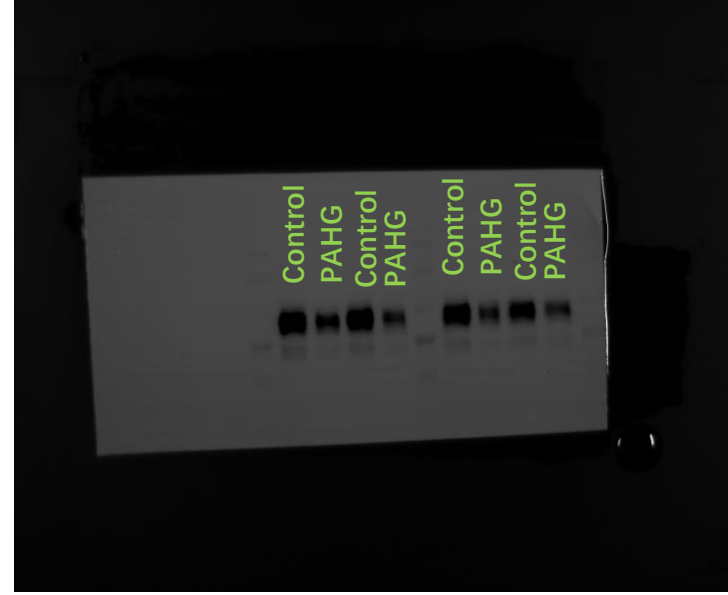

VCAM1

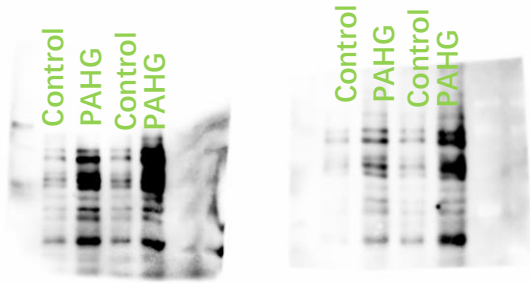

$\beta$ -actin

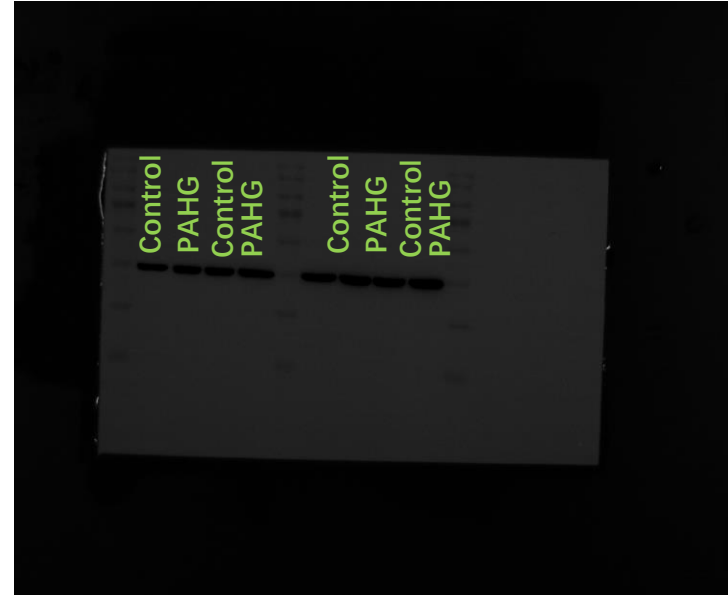

Supplement: Figure 1—figure supplement 1—source data 1. [file elife-97267-fig1-figsupp1-data1.zip › Figure 1-figure supplement 1-source data 1/Figure 1-figure supplement 1-Source data 1 Uncropped and labeled gels for Figure 1-supplement 1.pdf]

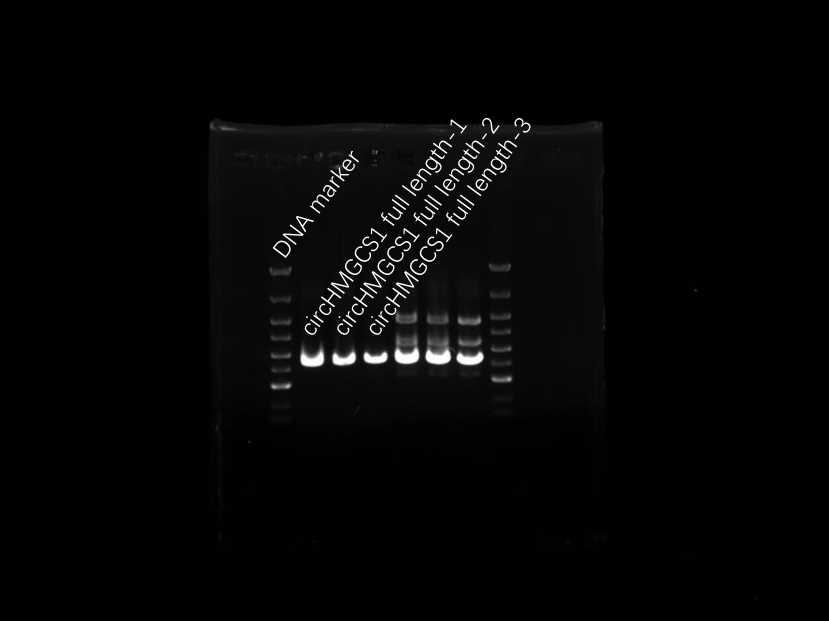

Supplement: Figure 1—figure supplement 1—source data 1. [file elife-97267-fig1-figsupp1-data1.zip › Figure 1-figure supplement 1-source data 1/Figure 1-figure supplement 1-Source data 2 Uncropped and labeled gels for Figure 1-supplement 1.pdf]

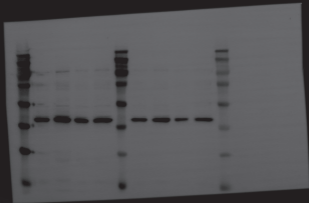

Supplement: Figure 1—figure supplement 1—source data 2. [file elife-97267-fig1-figsupp1-data2.zip › Figure 1-figure supplement 1-source data 2/Figure 1-figure supplement 1-source data 1 Raw unedited gels for Figure 1-figure supplement 1.pdf]

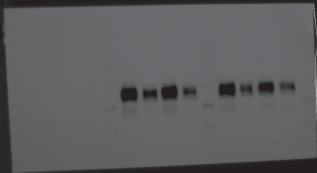

Supplement: Figure 1—figure supplement 1—source data 2. [file elife-97267-fig1-figsupp1-data2.zip › Figure 1-figure supplement 1-source data 2/Figure 1-figure supplement 1-source data 2 Raw unedited gels for Figure 1-figure supplement 1.pdf]

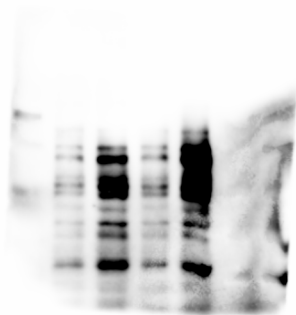

Supplement: Figure 1—figure supplement 1—source data 2. [file elife-97267-fig1-figsupp1-data2.zip › Figure 1-figure supplement 1-source data 2/Figure 1-figure supplement 1-source data 3 Raw unedited gels for Figure 1-figure supplement 1.pdf]

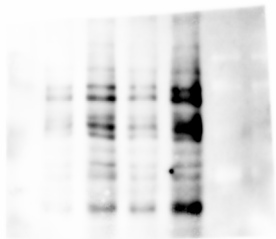

Supplement: Figure 1—figure supplement 1—source data 2. [file elife-97267-fig1-figsupp1-data2.zip › Figure 1-figure supplement 1-source data 2/Figure 1-figure supplement 1-source data 4 Raw unedited gels for Figure 1-figure supplement 1.pdf]

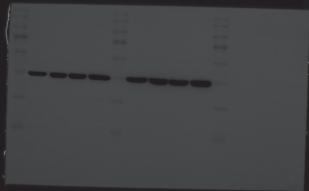

Supplement: Figure 1—figure supplement 1—source data 2. [file elife-97267-fig1-figsupp1-data2.zip › Figure 1-figure supplement 1-source data 2/Figure 1-figure supplement 1-source data 5 Raw unedited gels for Figure 1-figure supplement 1.pdf]

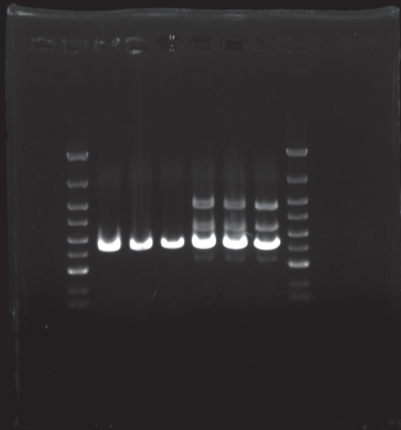

Supplement: Figure 1—figure supplement 1—source data 2. [file elife-97267-fig1-figsupp1-data2.zip › Figure 1-figure supplement 1-source data 2/Figure 1-figure supplement 1-source data 6 Raw unedited gels for Figure 1-figure supplement 1.pdf]

ET-1

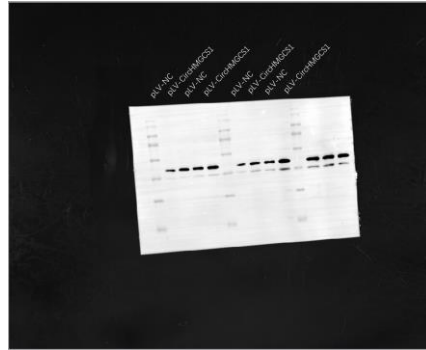

ICAM1

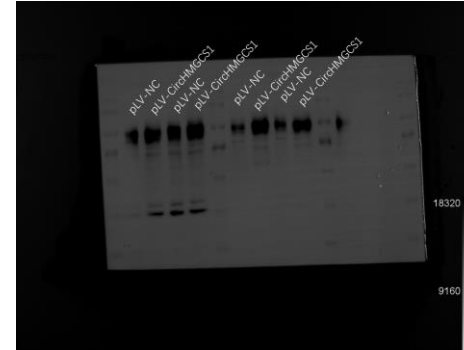

VCAM1

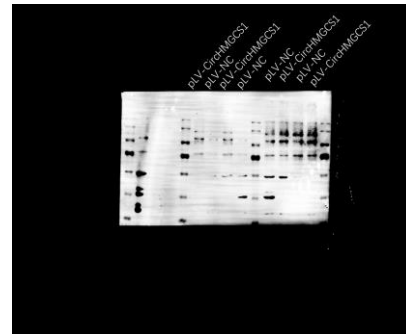

$\beta$ -actin

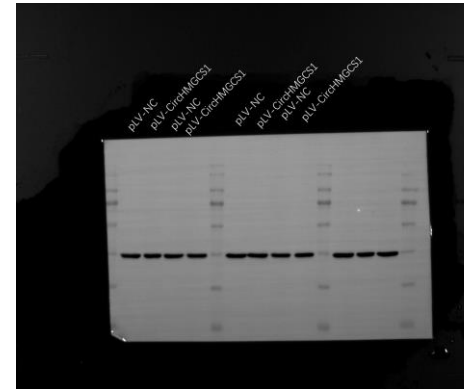

Supplement: Figure 2—source data 1. [file elife-97267-fig2-data1.zip › Figure 2-source data 1/Figure 2-Source data 1 Uncropped and labeled gels for Figure 2.pdf]

ET-1

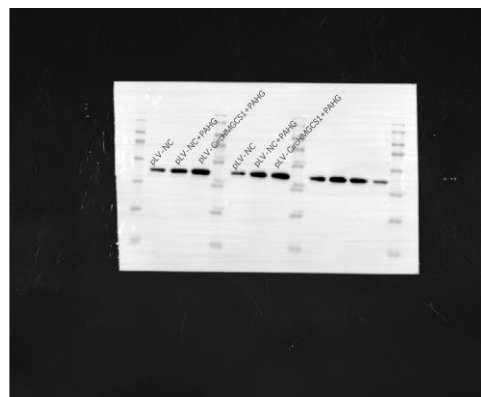

ICAM1

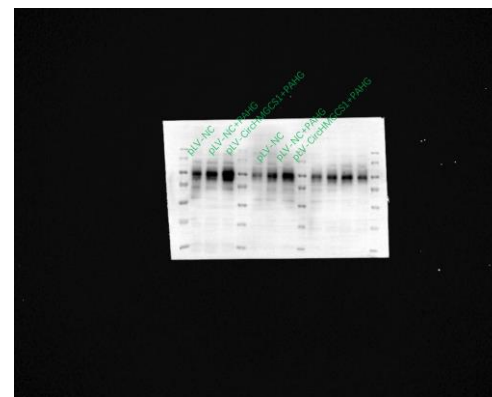

VCAM1

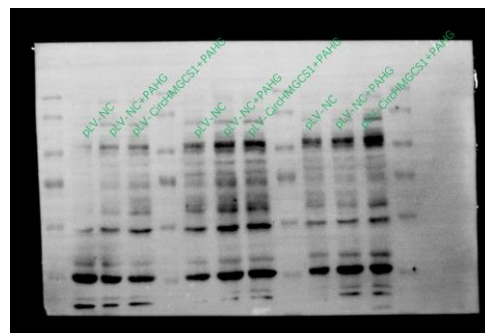

$\beta$ -actin

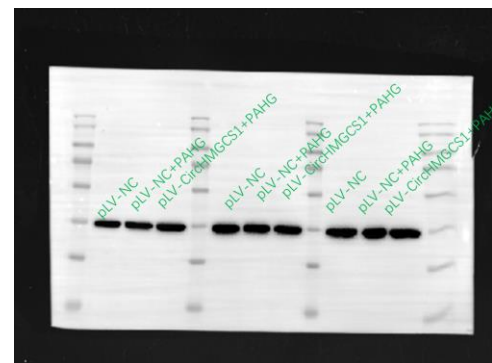

Supplement: Figure 2—source data 1. [file elife-97267-fig2-data1.zip › Figure 2-source data 1/Figure 2-Source data 2 Uncropped and labeled gels for Figure 2.pdf]

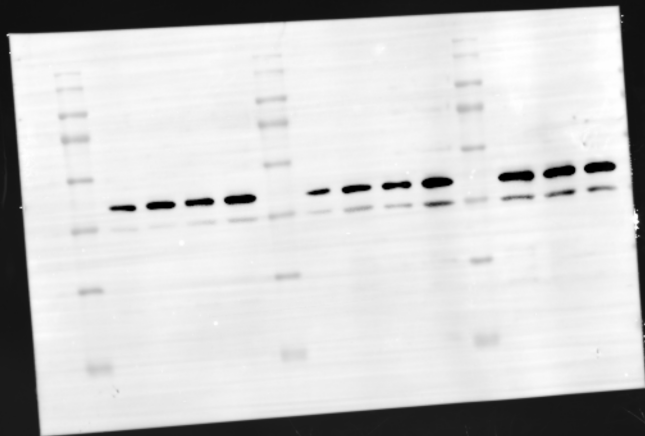

Supplement: Figure 2—source data 2. [file elife-97267-fig2-data2.zip › Figure 2-source data 2/Figure 2-source data 1 Raw unedited gels for Figure 2.pdf]

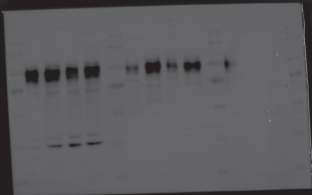

26065

13032

Supplement: Figure 2—source data 2. [file elife-97267-fig2-data2.zip › Figure 2-source data 2/Figure 2-source data 2 Raw unedited gels for Figure 2.pdf]

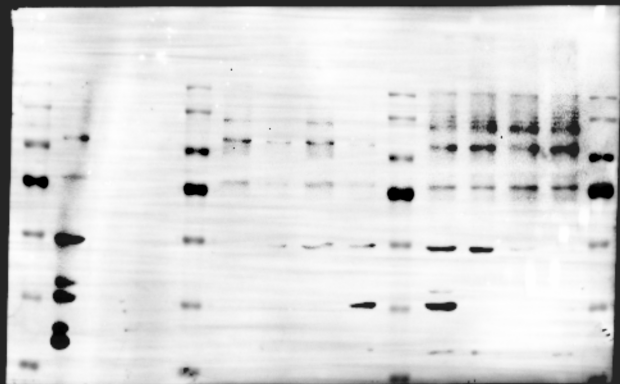

Supplement: Figure 2—source data 2. [file elife-97267-fig2-data2.zip › Figure 2-source data 2/Figure 2-source data 3 Raw unedited gels for Figure 2.pdf]

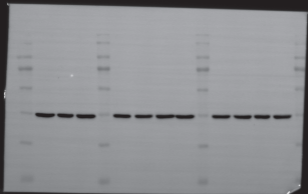

Supplement: Figure 2—source data 2. [file elife-97267-fig2-data2.zip › Figure 2-source data 2/Figure 2-source data 4 Raw unedited gels for Figure 2.pdf]

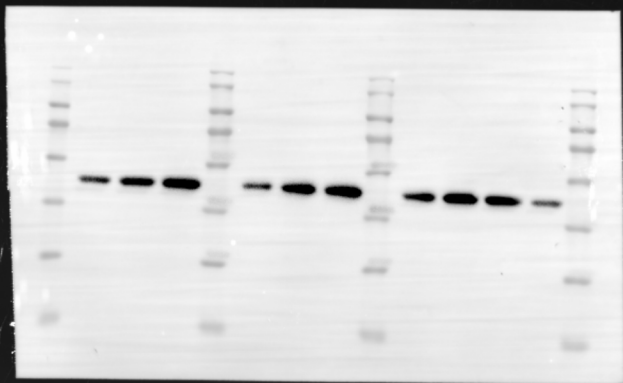

Supplement: Figure 2—source data 2. [file elife-97267-fig2-data2.zip › Figure 2-source data 2/Figure 2-source data 5 Raw unedited gels for Figure 2.pdf]

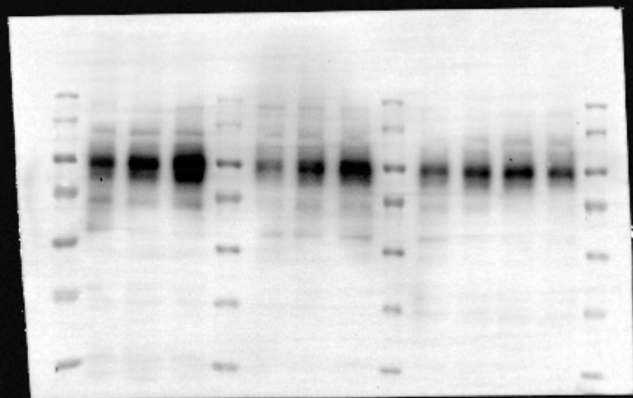

Supplement: Figure 2—source data 2. [file elife-97267-fig2-data2.zip › Figure 2-source data 2/Figure 2-source data 6 Raw unedited gels for Figure 2.pdf]

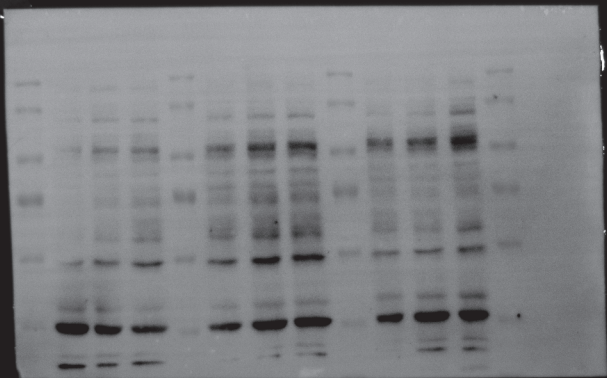

Supplement: Figure 2—source data 2. [file elife-97267-fig2-data2.zip › Figure 2-source data 2/Figure 2-source data 7 Raw unedited gels for Figure 2.pdf]

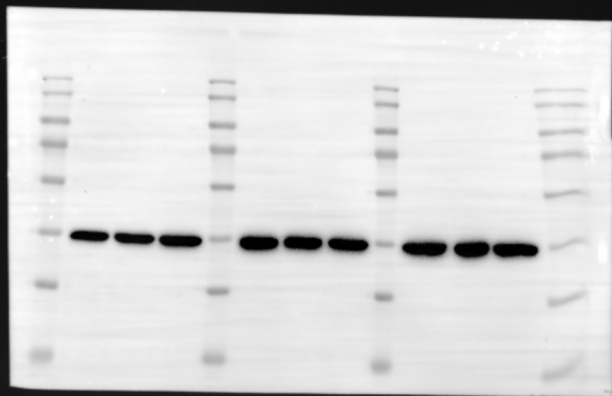

Supplement: Figure 2—source data 2. [file elife-97267-fig2-data2.zip › Figure 2-source data 2/Figure 2-source data 8 Raw unedited gels for Figure 2.pdf]

ET-1

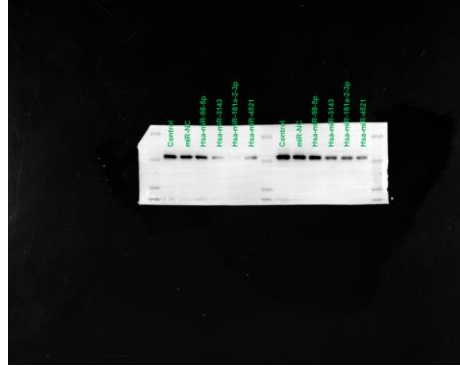

ICAM1

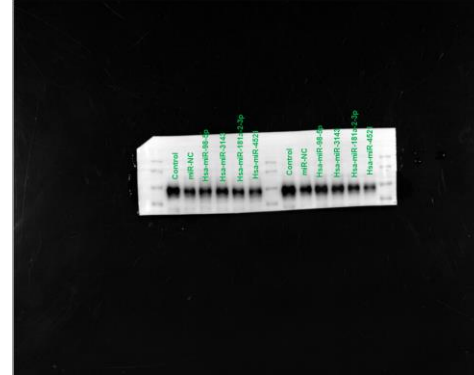

VCAM1

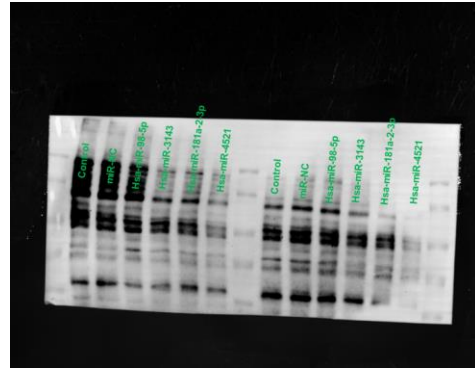

$\beta$ -actin

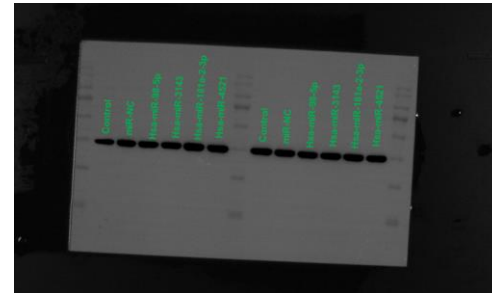

Supplement: Figure 3—source data 1. [file elife-97267-fig3-data1.zip › Figure 3-source data 1/Figure 3-Source data 1 Uncropped and labeled gels for Figure 3.pdf]

ET-1

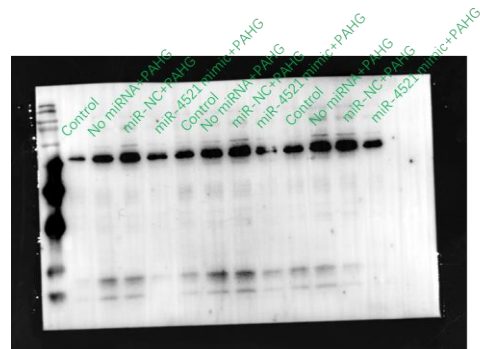

ICAM1

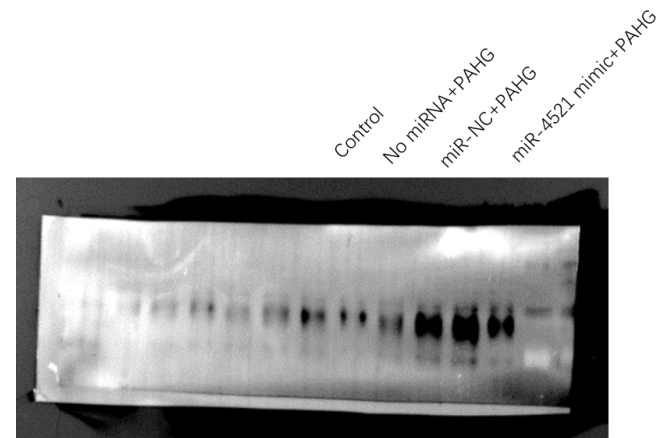

VCAM1

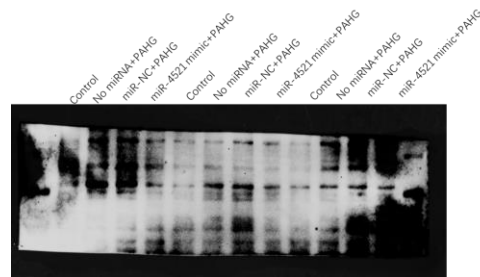

$\beta$ -actin

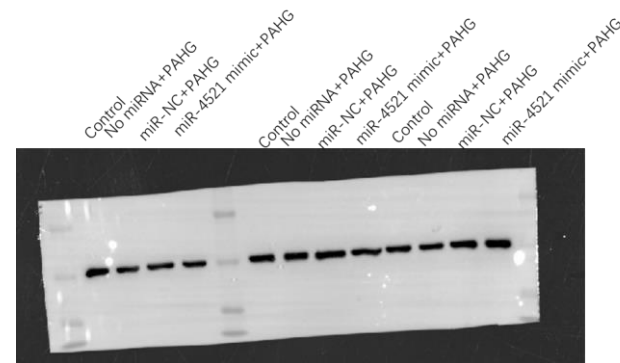

Supplement: Figure 3—source data 1. [file elife-97267-fig3-data1.zip › Figure 3-source data 1/Figure 3-Source data 2 Uncropped and labeled gels for Figure 3.pdf]

ET-1

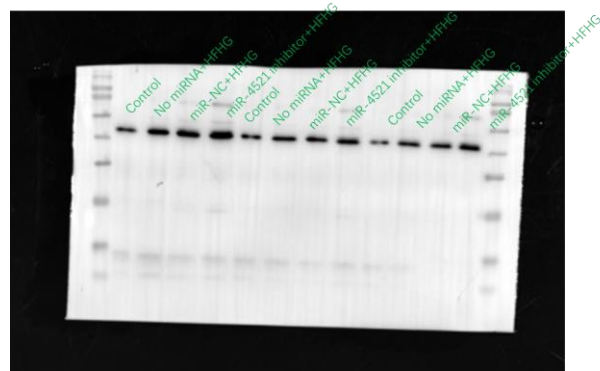

ICAM1

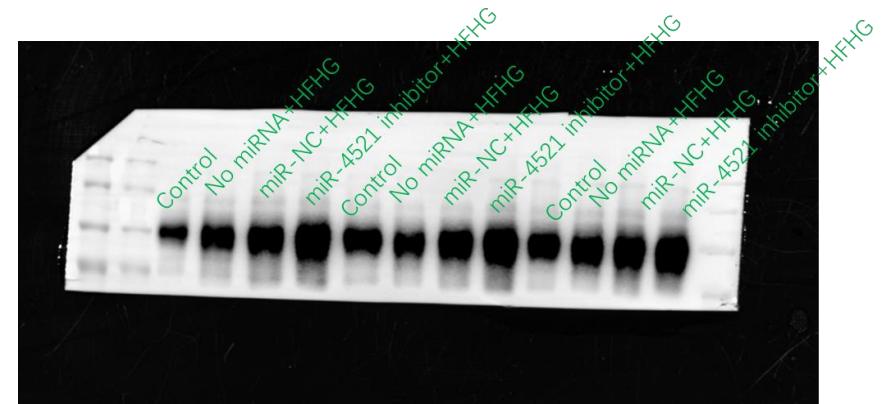

VCAM1

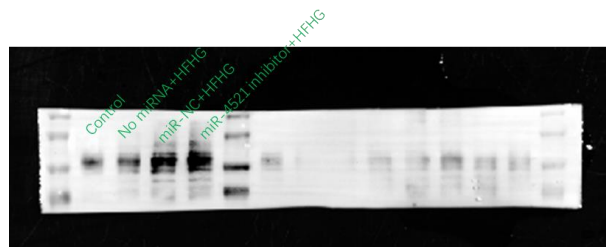

$\beta$ -actin

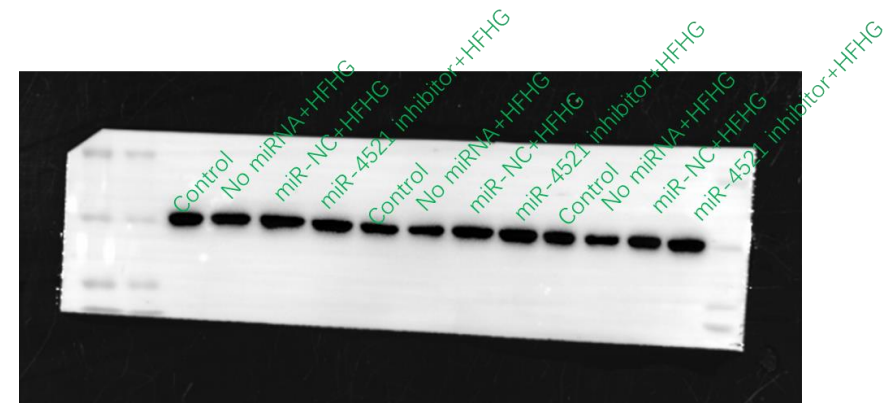

Supplement: Figure 3—source data 1. [file elife-97267-fig3-data1.zip › Figure 3-source data 1/Figure 3-Source data 3 Uncropped and labeled gels for Figure 3.pdf]

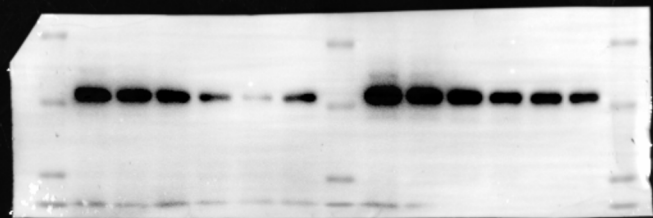

Supplement: Figure 3—source data 2. [file elife-97267-fig3-data2.zip › Figure 3-source data 2/Figure 3-source data 1 Raw unedited gels for Figure 3.pdf]

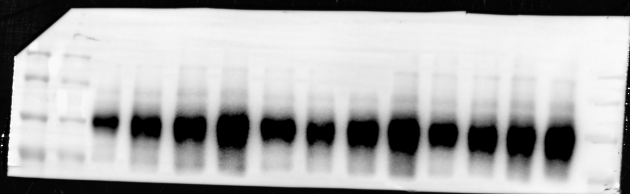

Supplement: Figure 3—source data 2. [file elife-97267-fig3-data2.zip › Figure 3-source data 2/Figure 3-source data 10 Raw unedited gels for Figure 3.pdf]

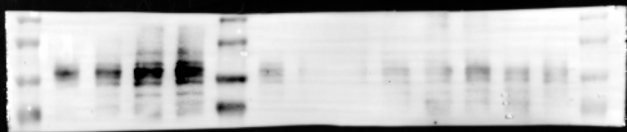

Supplement: Figure 3—source data 2. [file elife-97267-fig3-data2.zip › Figure 3-source data 2/Figure 3-source data 11 Raw unedited gels for Figure 3.pdf]

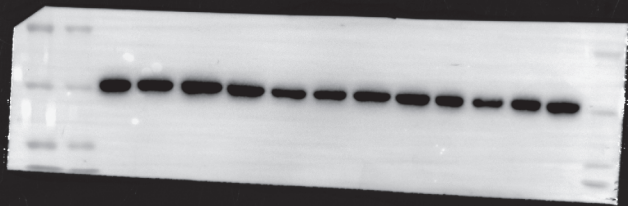

Supplement: Figure 3—source data 2. [file elife-97267-fig3-data2.zip › Figure 3-source data 2/Figure 3-source data 12 Raw unedited gels for Figure 3.pdf]

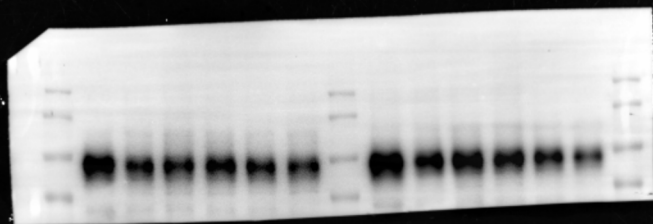

Supplement: Figure 3—source data 2. [file elife-97267-fig3-data2.zip › Figure 3-source data 2/Figure 3-source data 2 Raw unedited gels for Figure 3.pdf]

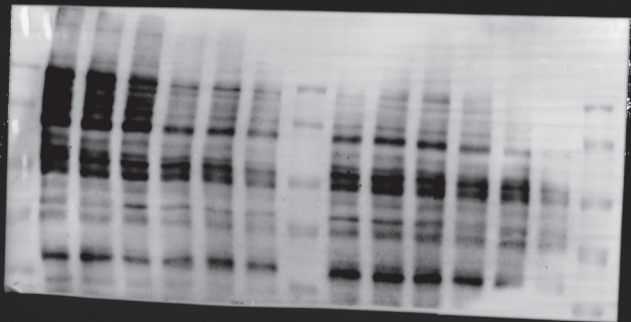

Supplement: Figure 3—source data 2. [file elife-97267-fig3-data2.zip › Figure 3-source data 2/Figure 3-source data 3 Raw unedited gels for Figure 3.pdf]

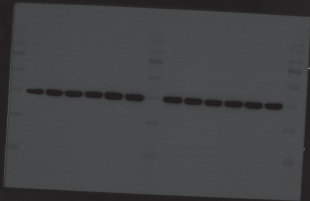

Supplement: Figure 3—source data 2. [file elife-97267-fig3-data2.zip › Figure 3-source data 2/Figure 3-source data 4 Raw unedited gels for Figure 3.pdf]

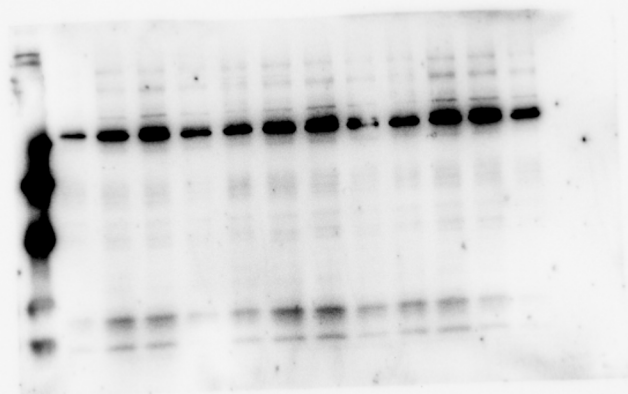

Supplement: Figure 3—source data 2. [file elife-97267-fig3-data2.zip › Figure 3-source data 2/Figure 3-source data 5 Raw unedited gels for Figure 3.pdf]

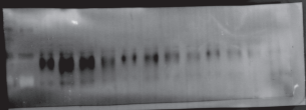

Supplement: Figure 3—source data 2. [file elife-97267-fig3-data2.zip › Figure 3-source data 2/Figure 3-source data 6 Raw unedited gels for Figure 3.pdf]

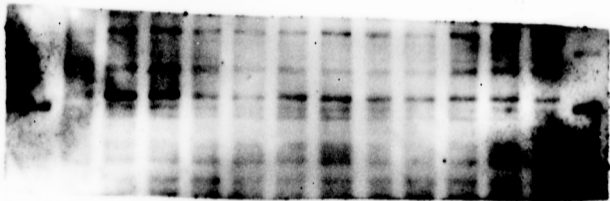

Supplement: Figure 3—source data 2. [file elife-97267-fig3-data2.zip › Figure 3-source data 2/Figure 3-source data 7 Raw unedited gels for Figure 3.pdf]

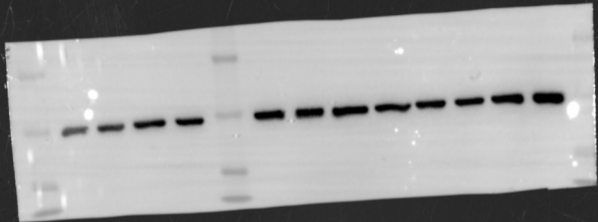

Supplement: Figure 3—source data 2. [file elife-97267-fig3-data2.zip › Figure 3-source data 2/Figure 3-source data 8 Raw unedited gels for Figure 3.pdf]

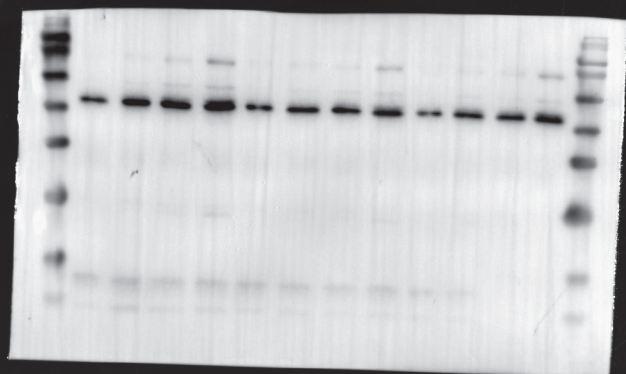

Supplement: Figure 3—source data 2. [file elife-97267-fig3-data2.zip › Figure 3-source data 2/Figure 3-source data 9 Raw unedited gels for Figure 3.pdf]

ET-1

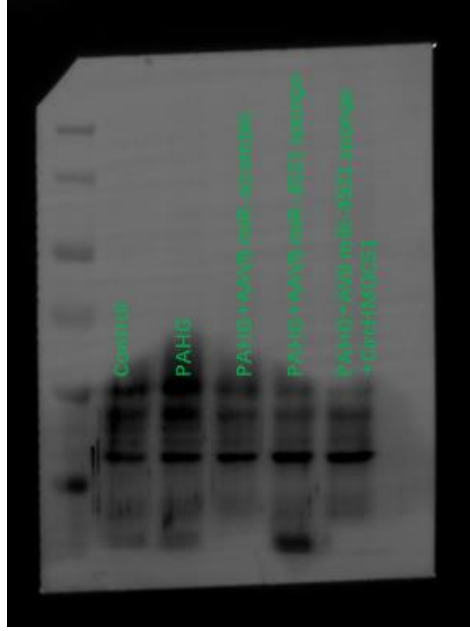

ICAM1

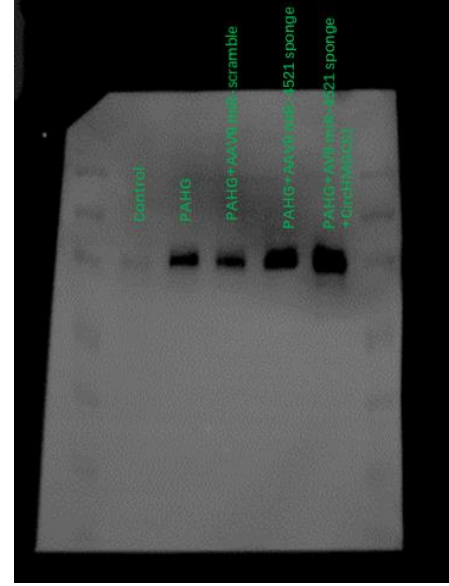

VCAM1

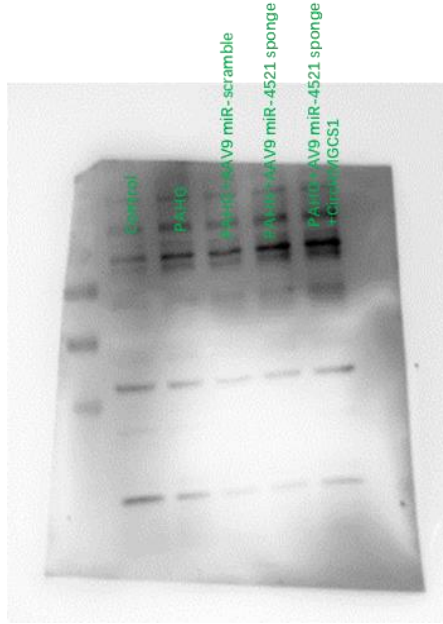

$\beta$ -actin

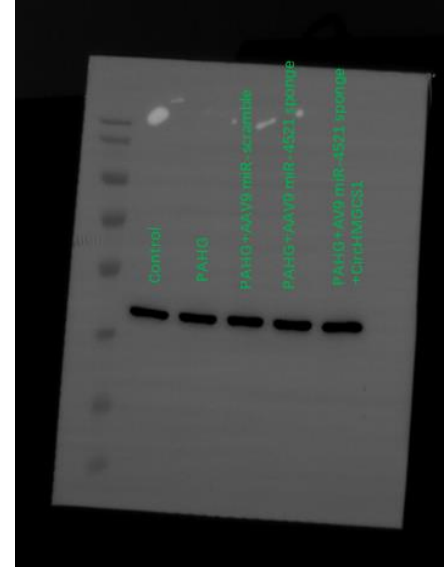

Supplement: Figure 4—source data 1. [file elife-97267-fig4-data1.zip › Figure 4-source data 1/Figure 4-Source data 1 Uncropped and labeled gels for Figure 4.pdf]

ET-1

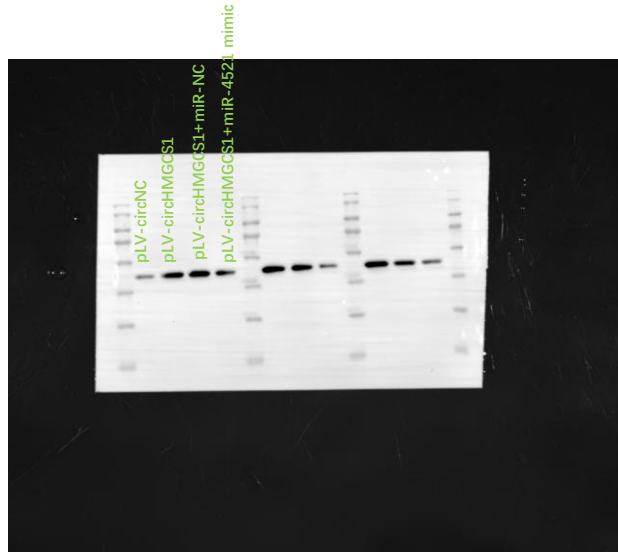

ICAM1

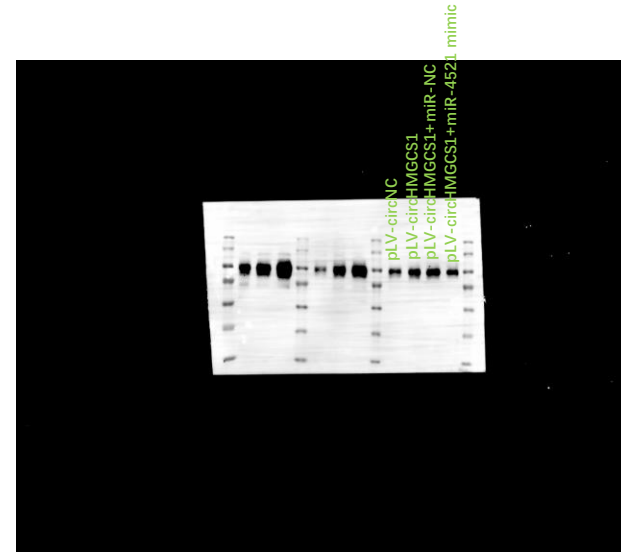

VCAM1

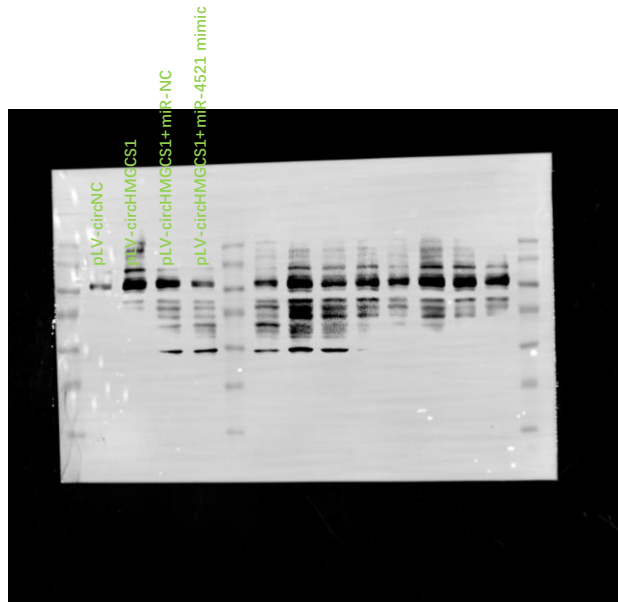

$\beta$ -actin

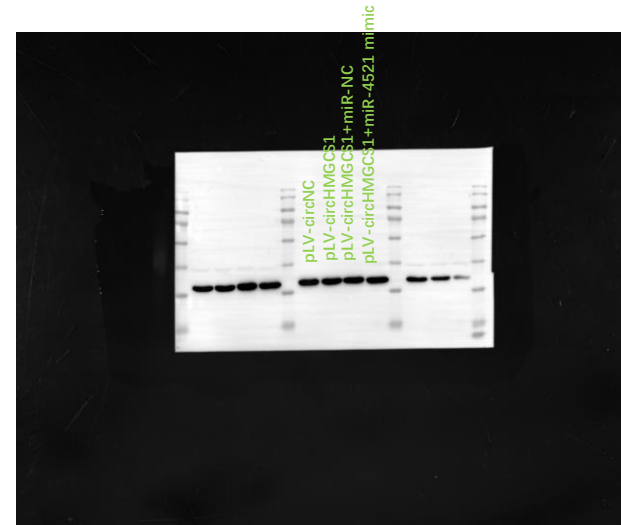

Supplement: Figure 4—source data 1. [file elife-97267-fig4-data1.zip › Figure 4-source data 1/Figure 4-Source data 2 Uncropped and labeled gels for Figure 4.pdf]

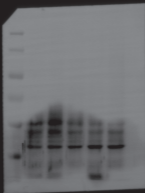

Supplement: Figure 4—source data 2. [file elife-97267-fig4-data2.zip › Figure 4-source data 2/Figure 4-source data 1 Raw unedited gels for Figure 4.pdf]

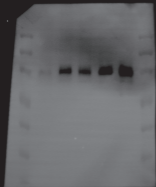

Supplement: Figure 4—source data 2. [file elife-97267-fig4-data2.zip › Figure 4-source data 2/Figure 4-source data 2 Raw unedited gels for Figure 4.pdf]

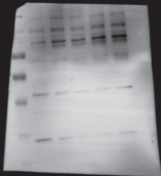

Supplement: Figure 4—source data 2. [file elife-97267-fig4-data2.zip › Figure 4-source data 2/Figure 4-source data 3 Raw unedited gels for Figure 4.pdf]

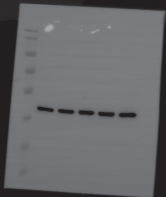

Supplement: Figure 4—source data 2. [file elife-97267-fig4-data2.zip › Figure 4-source data 2/Figure 4-source data 4 Raw unedited gels for Figure 4.pdf]

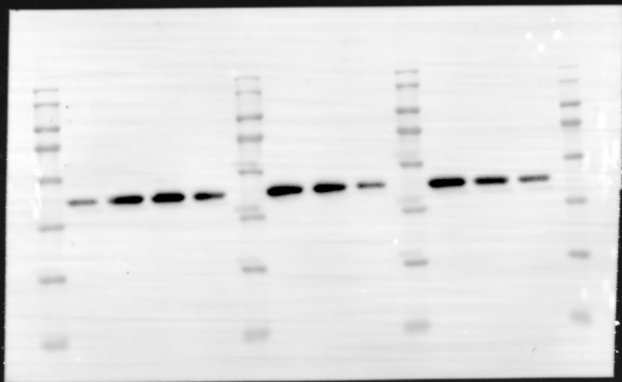

Supplement: Figure 4—source data 2. [file elife-97267-fig4-data2.zip › Figure 4-source data 2/Figure 4-source data 5 Raw unedited gels for Figure 4.pdf]

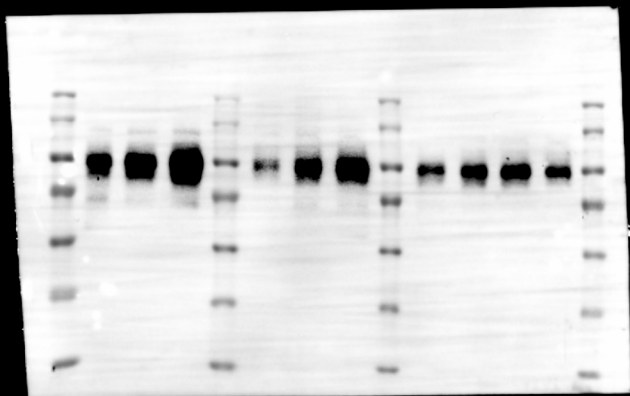

Supplement: Figure 4—source data 2. [file elife-97267-fig4-data2.zip › Figure 4-source data 2/Figure 4-source data 6 Raw unedited gels for Figure 4.pdf]

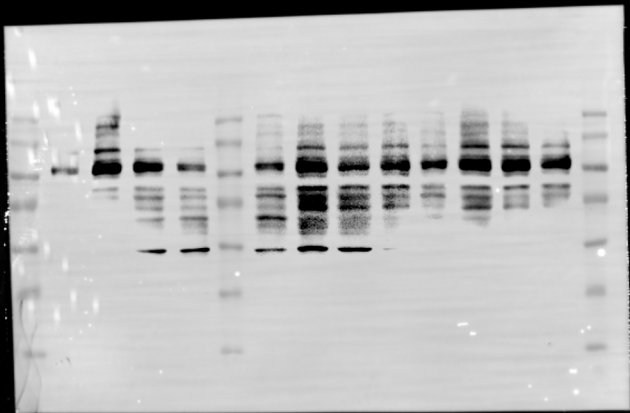

Supplement: Figure 4—source data 2. [file elife-97267-fig4-data2.zip › Figure 4-source data 2/Figure 4-source data 7 Raw unedited gels for Figure 4.pdf]

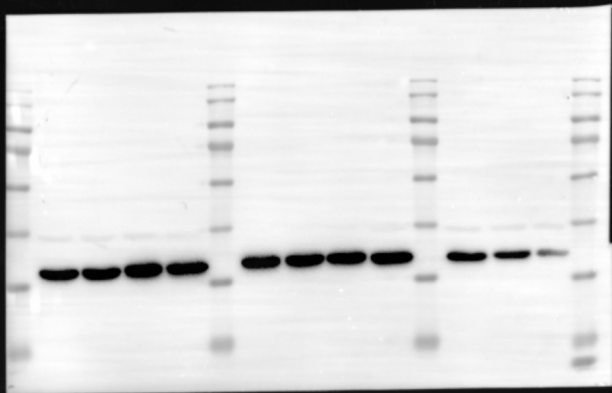

Supplement: Figure 4—source data 2. [file elife-97267-fig4-data2.zip › Figure 4-source data 2/Figure 4-source data 8 Raw unedited gels for Figure 4.pdf]

Input  
Biotin-circHMGCs1-MUT  
Biotin-circHMGCs1-WT

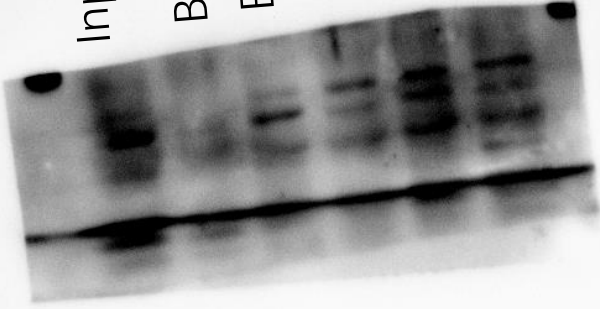

Supplement: Figure 4—figure supplement 1—source data 1. [file elife-97267-fig4-figsupp1-data1.zip › Figure 4-figure supplement 1-source data 1/Figure 4-figure supplement 1-Source data 1 Uncropped and labeled gels for Figure 4-supplement 1.pdf]

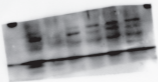

Supplement: Figure 4—figure supplement 1—source data 2. [file elife-97267-fig4-figsupp1-data2.zip › Figure 4-figure supplement 1-source data 2/Figure 4-figure supplement 1-source data 1 Raw unedited gels for Figure 4-figure supplement 1.pdf]

ET-1

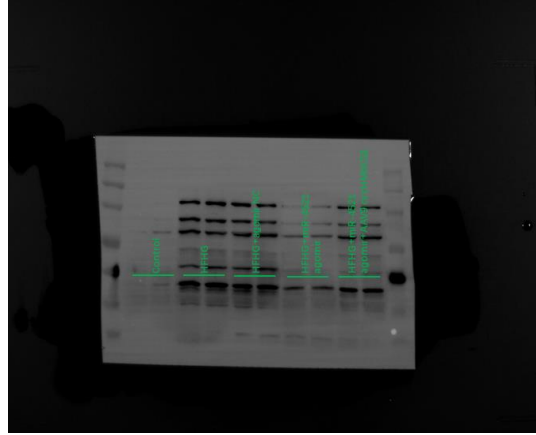

ICAM1

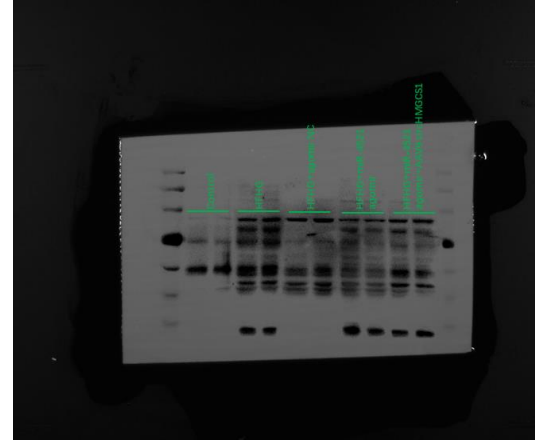

VCAM1

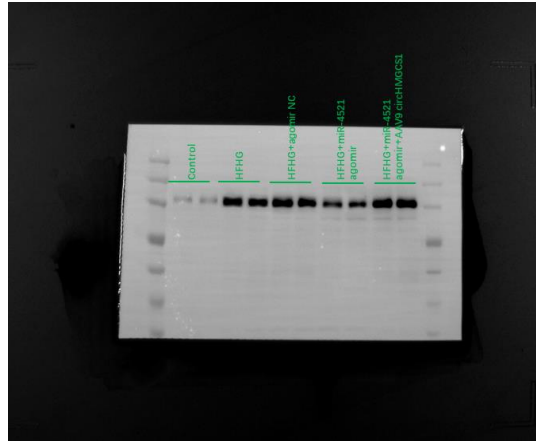

$\beta$ -actin

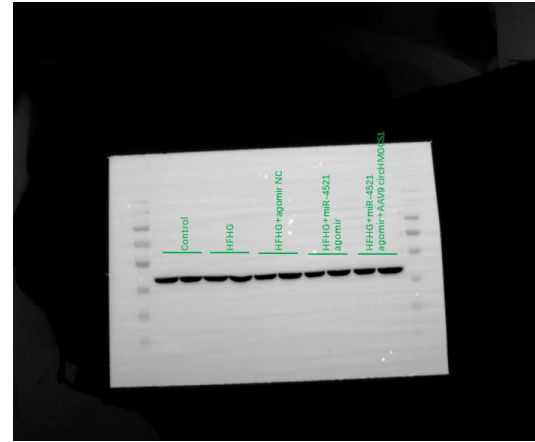

Supplement: Figure 5—source data 1. [file elife-97267-fig5-data1.zip › Figure 5-source data 1/Figure 5-Source data 1 Uncropped and labeled gels for Figure 5.pdf]

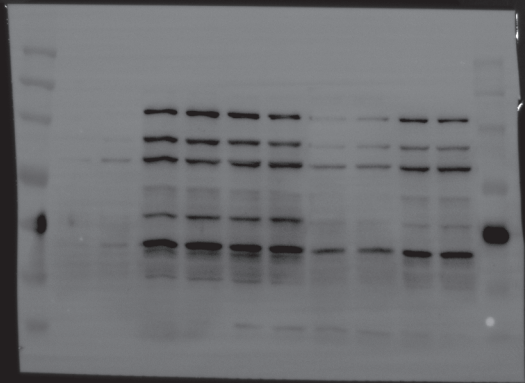

Supplement: Figure 5—source data 2. [file elife-97267-fig5-data2.zip › Figure 5-source data 2/Figure 5-source data 1 Raw unedited gels for Figure 5.pdf]

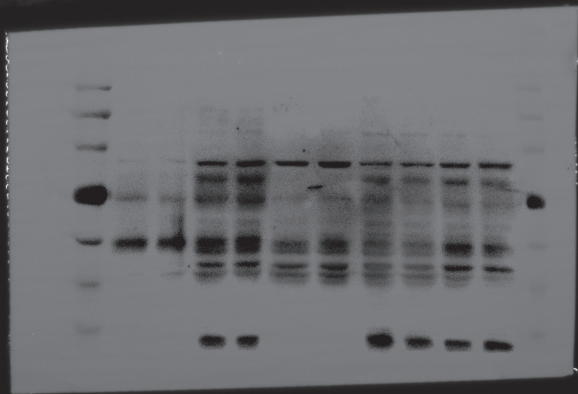

Supplement: Figure 5—source data 2. [file elife-97267-fig5-data2.zip › Figure 5-source data 2/Figure 5-source data 2 Raw unedited gels for Figure 5.pdf]

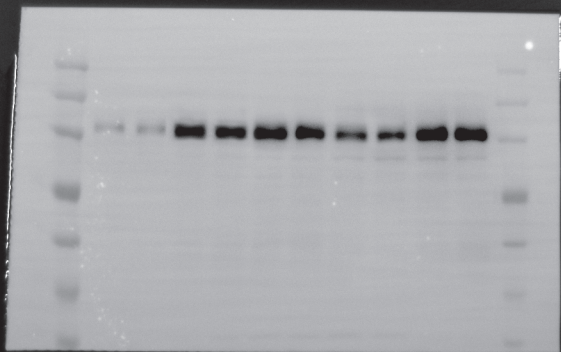

Supplement: Figure 5—source data 2. [file elife-97267-fig5-data2.zip › Figure 5-source data 2/Figure 5-source data 3 Raw unedited gels for Figure 5.pdf]

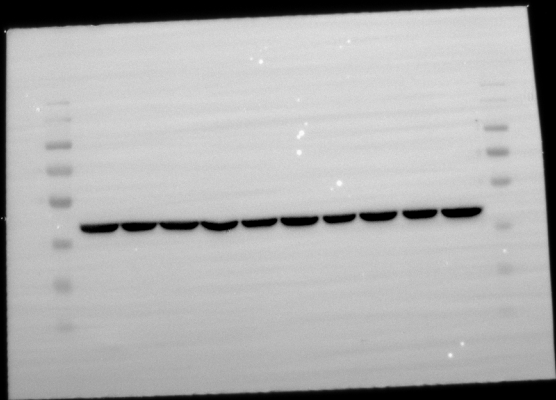

Supplement: Figure 5—source data 2. [file elife-97267-fig5-data2.zip › Figure 5-source data 2/Figure 5-source data 4 Raw unedited gels for Figure 5.pdf]

ARG1

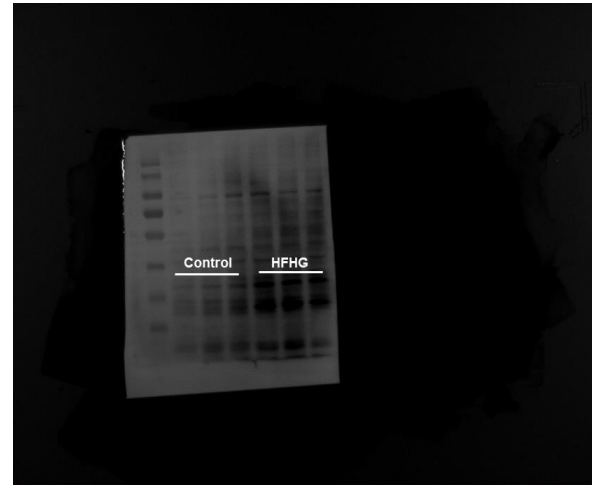

$\beta$ -actin

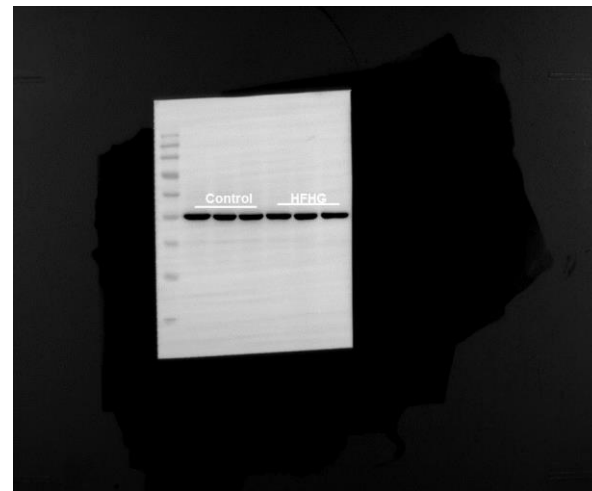

Supplement: Figure 6—source data 1. [file elife-97267-fig6-data1.zip › Figure 6-source data 1/Figure 6-Source data 1 Uncropped and labeled gels for Figure 6.pdf]

ET-1

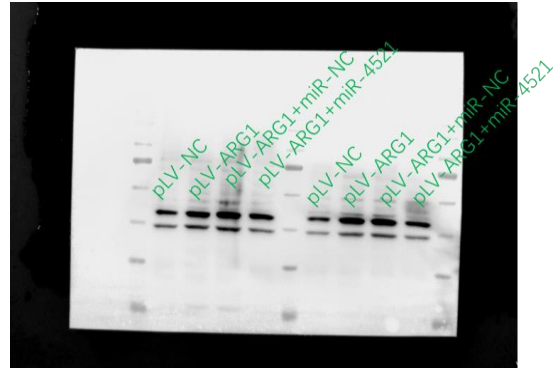

ICAM1

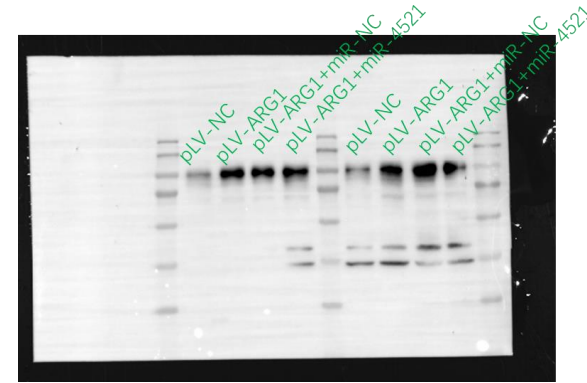

VCAM1

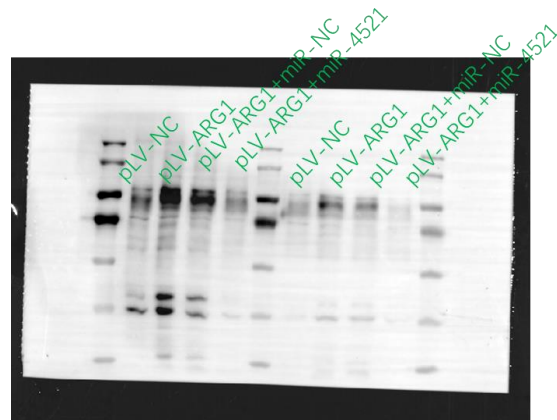

actin

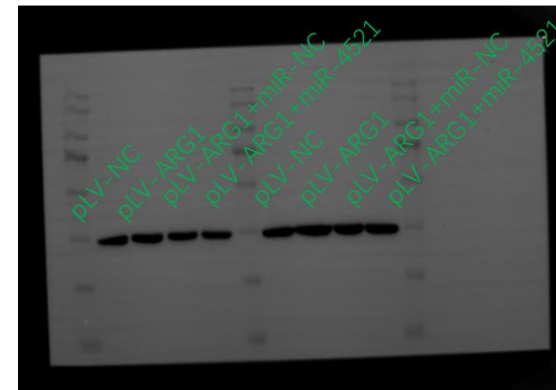

Supplement: Figure 6—source data 1. [file elife-97267-fig6-data1.zip › Figure 6-source data 1/Figure 6-Source data 2 Uncropped and labeled gels for Figure 6.pdf]

actin

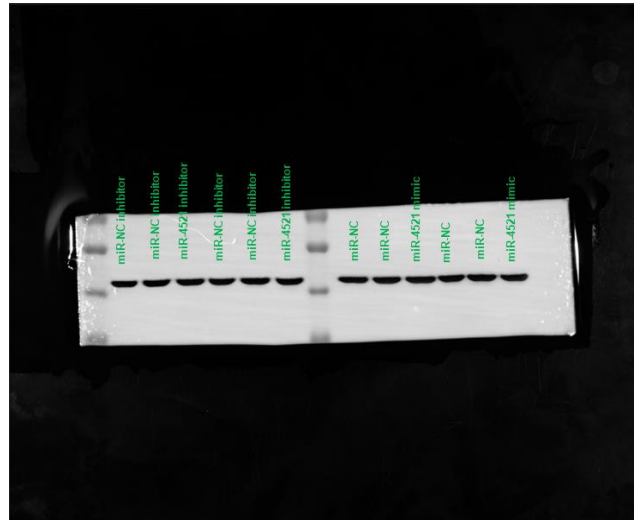

ARG1

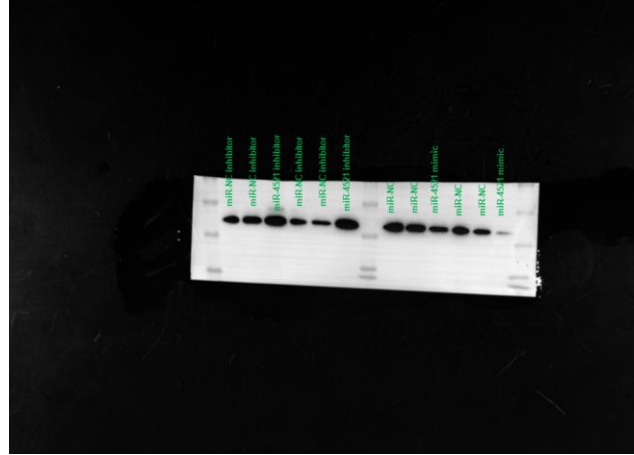

Supplement: Figure 6—source data 1. [file elife-97267-fig6-data1.zip › Figure 6-source data 1/Figure 6-Source data 3 Uncropped and labeled gels for Figure 6.pdf]

ARG1

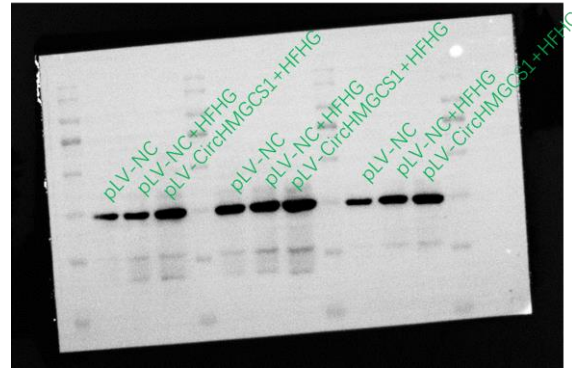

$\beta$ -actin

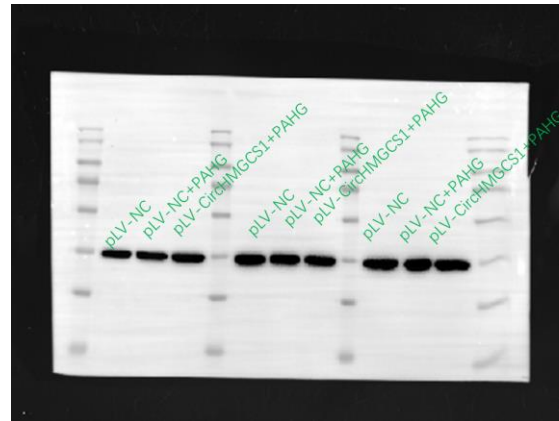

Supplement: Figure 6—source data 1. [file elife-97267-fig6-data1.zip › Figure 6-source data 1/Figure 6-Source data 4 Uncropped and labeled gels for Figure 6.pdf]

ARG1

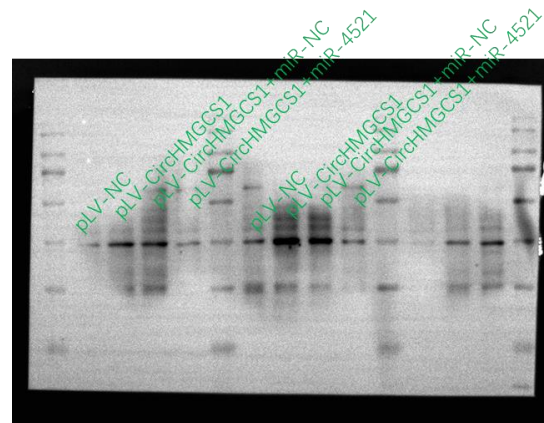

$\beta$ -actin

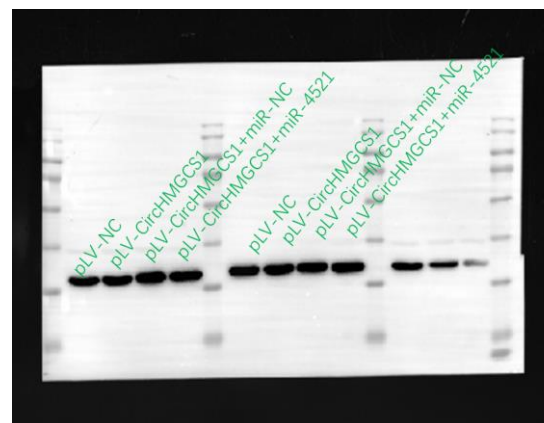

Supplement: Figure 6—source data 1. [file elife-97267-fig6-data1.zip › Figure 6-source data 1/Figure 6-Source data 5 Uncropped and labeled gels for Figure 6.pdf]

$\beta$ -actin

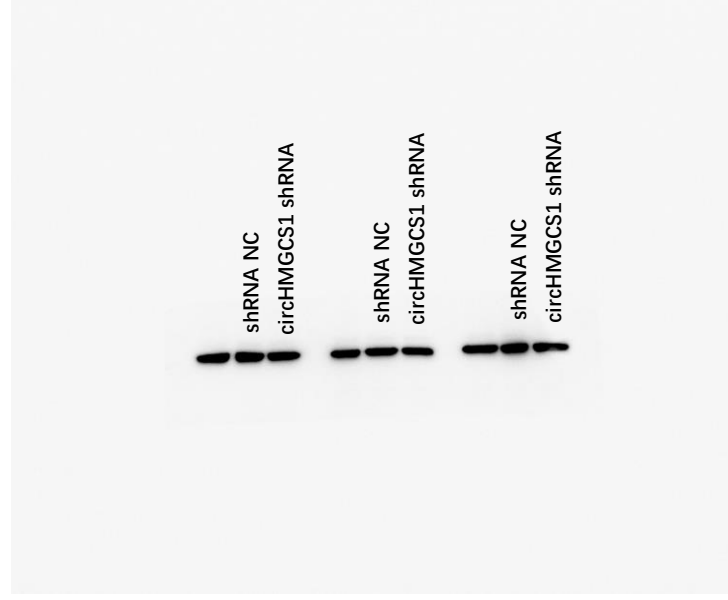

ARG1

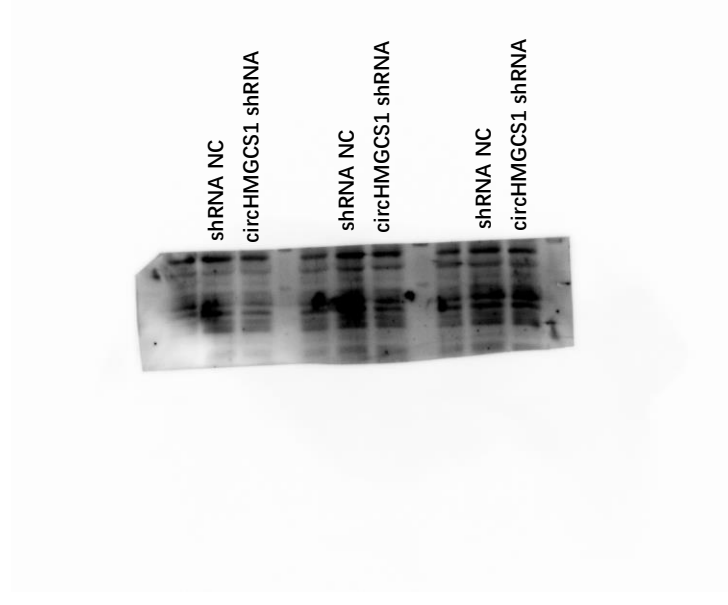

Supplement: Figure 6—source data 1. [file elife-97267-fig6-data1.zip › Figure 6-source data 1/Figure 6-Source data 6 Uncropped and labeled gels for Figure 6.pdf]

ARG1

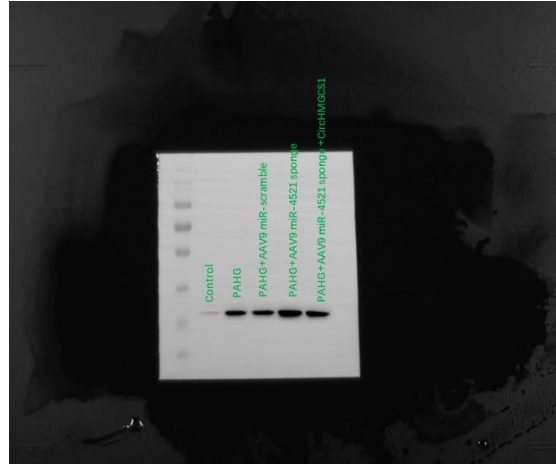

$\beta$ -actin

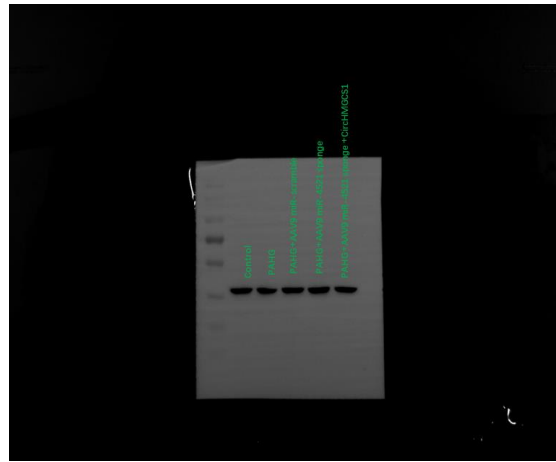

Supplement: Figure 6—source data 1. [file elife-97267-fig6-data1.zip › Figure 6-source data 1/Figure 6-Source data 7 Uncropped and labeled gels for Figure 6.pdf]

ARG1

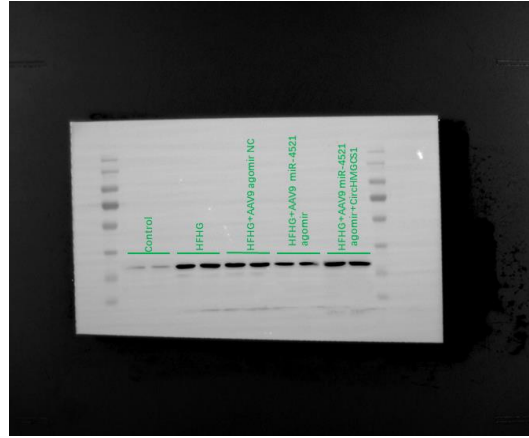

$\beta$ -actin

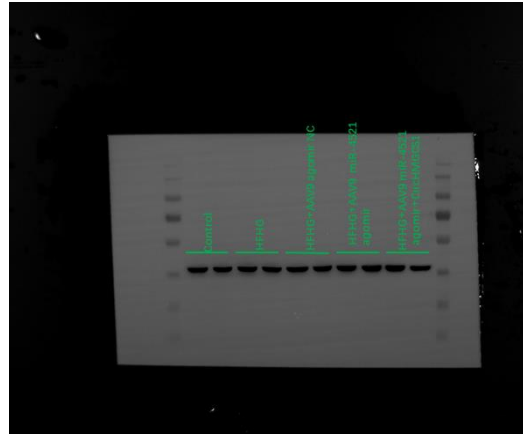

Supplement: Figure 6—source data 1. [file elife-97267-fig6-data1.zip › Figure 6-source data 1/Figure 6-Source data 8 Uncropped and labeled gels for Figure 6.pdf]

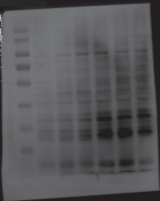

Supplement: Figure 6—source data 2. [file elife-97267-fig6-data2.zip › Figure 6-source data 2/Figure 6-source data 1 Raw unedited gels for Figure 6.pdf]

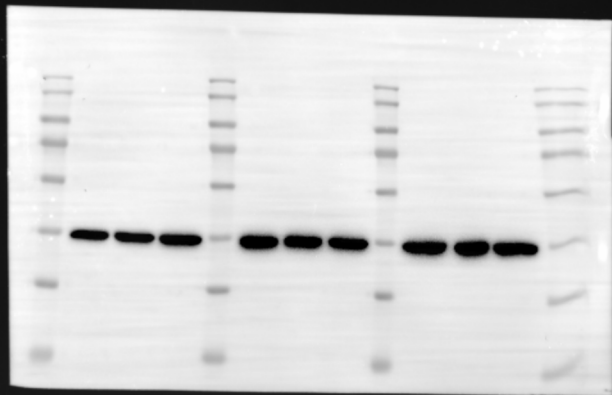

Supplement: Figure 6—source data 2. [file elife-97267-fig6-data2.zip › Figure 6-source data 2/Figure 6-source data 10 Raw unedited gels for Figure 6.pdf]

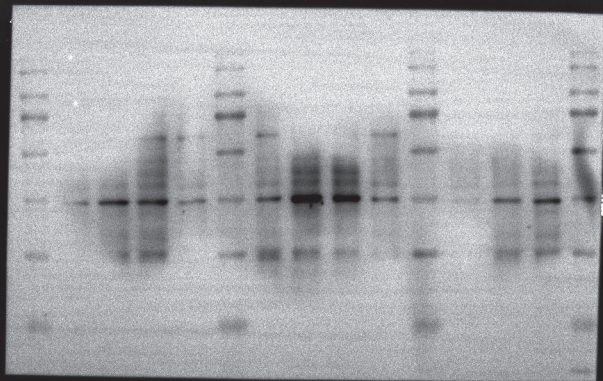

2958

6817

Supplement: Figure 6—source data 2. [file elife-97267-fig6-data2.zip › Figure 6-source data 2/Figure 6-source data 11 Raw unedited gels for Figure 6.pdf]

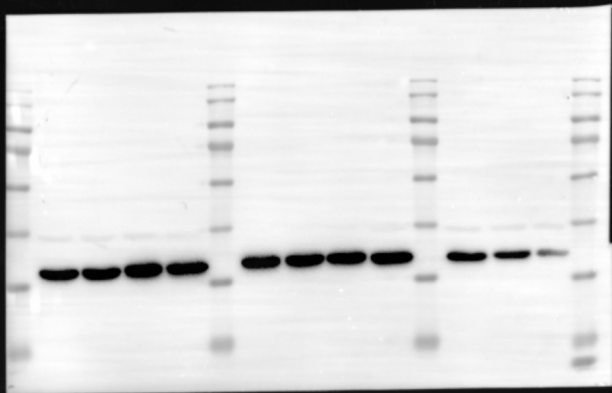

Supplement: Figure 6—source data 2. [file elife-97267-fig6-data2.zip › Figure 6-source data 2/Figure 6-source data 12 Raw unedited gels for Figure 6.pdf]

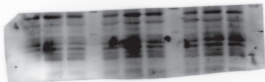

Supplement: Figure 6—source data 2. [file elife-97267-fig6-data2.zip › Figure 6-source data 2/Figure 6-source data 13 Raw unedited gels for Figure 6.pdf]

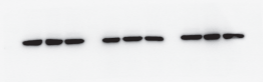

Supplement: Figure 6—source data 2. [file elife-97267-fig6-data2.zip › Figure 6-source data 2/Figure 6-source data 14 Raw unedited gels for Figure 6.pdf]

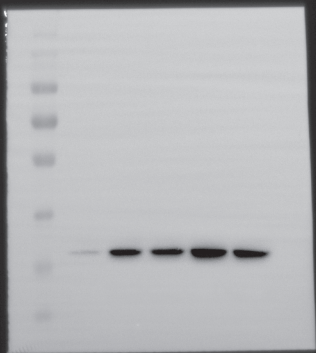

Supplement: Figure 6—source data 2. [file elife-97267-fig6-data2.zip › Figure 6-source data 2/Figure 6-source data 15 Raw unedited gels for Figure 6.pdf]

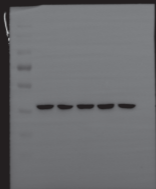

Supplement: Figure 6—source data 2. [file elife-97267-fig6-data2.zip › Figure 6-source data 2/Figure 6-source data 16 Raw unedited gels for Figure 6.pdf]

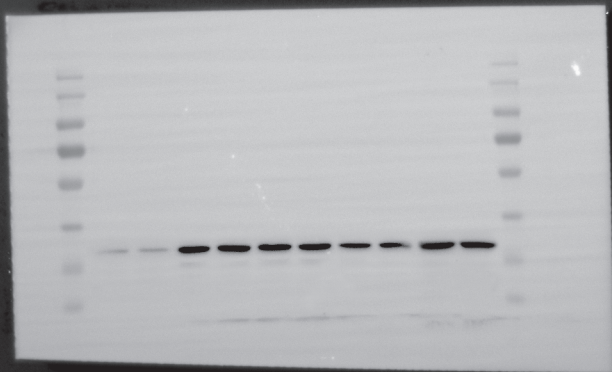

Supplement: Figure 6—source data 2. [file elife-97267-fig6-data2.zip › Figure 6-source data 2/Figure 6-source data 17 Raw unedited gels for Figure 6.pdf]

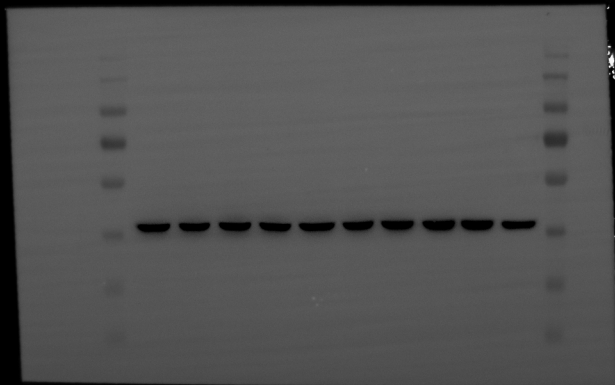

Supplement: Figure 6—source data 2. [file elife-97267-fig6-data2.zip › Figure 6-source data 2/Figure 6-source data 18 Raw unedited gels for Figure 6.pdf]

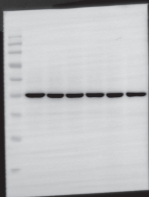

Supplement: Figure 6—source data 2. [file elife-97267-fig6-data2.zip › Figure 6-source data 2/Figure 6-source data 2 Raw unedited gels for Figure 6.pdf]

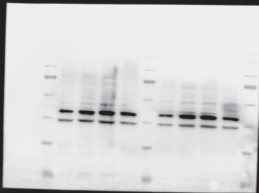

Supplement: Figure 6—source data 2. [file elife-97267-fig6-data2.zip › Figure 6-source data 2/Figure 6-source data 3 Raw unedited gels for Figure 6.pdf]

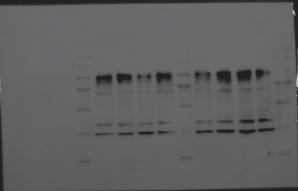

Supplement: Figure 6—source data 2. [file elife-97267-fig6-data2.zip › Figure 6-source data 2/Figure 6-source data 4 Raw unedited gels for Figure 6.pdf]

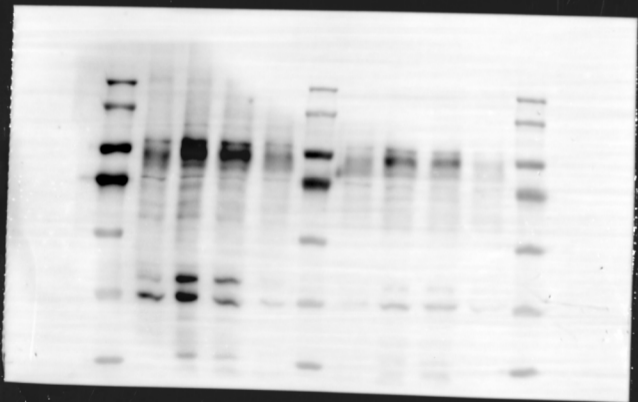

Supplement: Figure 6—source data 2. [file elife-97267-fig6-data2.zip › Figure 6-source data 2/Figure 6-source data 5 Raw unedited gels for Figure 6.pdf]

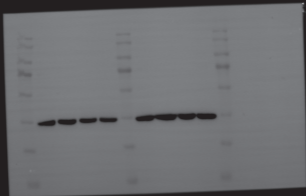

Supplement: Figure 6—source data 2. [file elife-97267-fig6-data2.zip › Figure 6-source data 2/Figure 6-source data 6 Raw unedited gels for Figure 6.pdf]

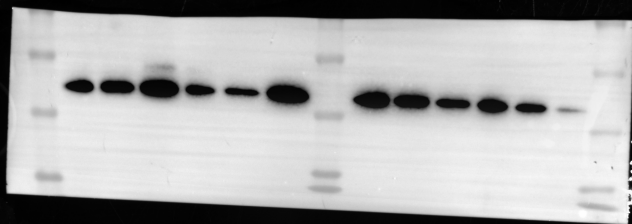

Supplement: Figure 6—source data 2. [file elife-97267-fig6-data2.zip › Figure 6-source data 2/Figure 6-source data 7 Raw unedited gels for Figure 6.pdf]

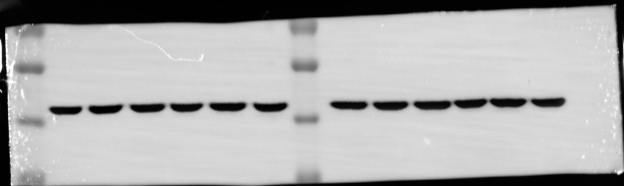

Supplement: Figure 6—source data 2. [file elife-97267-fig6-data2.zip › Figure 6-source data 2/Figure 6-source data 8 Raw unedited gels for Figure 6.pdf]

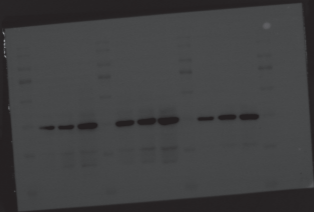

Supplement: Figure 6—source data 2. [file elife-97267-fig6-data2.zip › Figure 6-source data 2/Figure 6-source data 9 Raw unedited gels for Figure 6.pdf]

ARG1

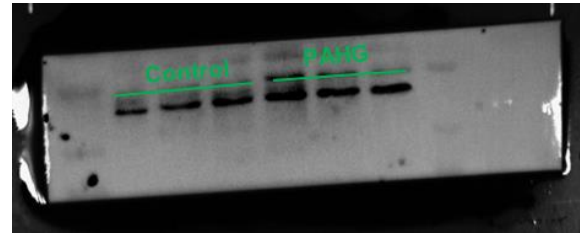

ARG2

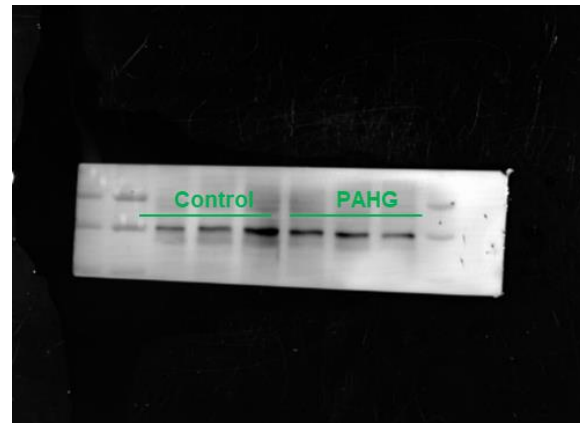

$\beta$ -actin

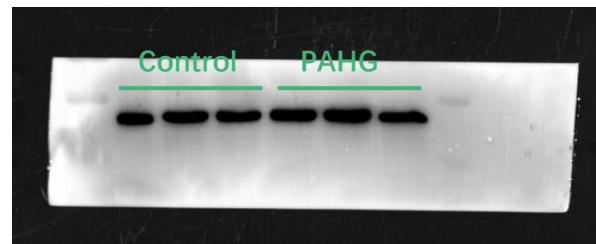

Supplement: Figure 6—figure supplement 1—source data 1. [file elife-97267-fig6-figsupp1-data1.zip › Figure 6-figure supplement 1-source data 1/Figure 6-figure supplement 1-Source data 1 Uncropped and labeled gels for Figure 6-supplement 1.pdf]

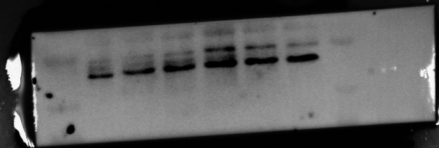

Supplement: Figure 6—figure supplement 1—source data 2. [file elife-97267-fig6-figsupp1-data2.zip › Figure 6-figure supplement 1-source data 2/Figure 6-figure supplement 1-source data 1 Raw unedited gels for Figure 6-figure supplement 1.pdf]

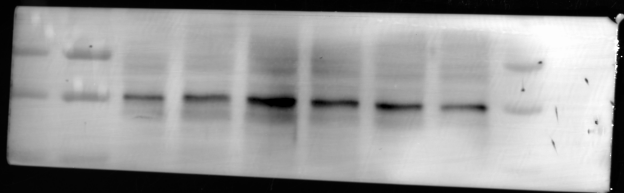

Supplement: Figure 6—figure supplement 1—source data 2. [file elife-97267-fig6-figsupp1-data2.zip › Figure 6-figure supplement 1-source data 2/Figure 6-figure supplement 1-source data 2 Raw unedited gels for Figure 6-figure supplement 1.pdf]

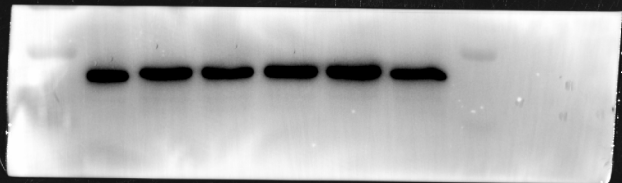

Supplement: Figure 6—figure supplement 1—source data 2. [file elife-97267-fig6-figsupp1-data2.zip › Figure 6-figure supplement 1-source data 2/Figure 6-figure supplement 1-source data 3 Raw unedited gels for Figure 6-figure supplement 1.pdf]

ET-1

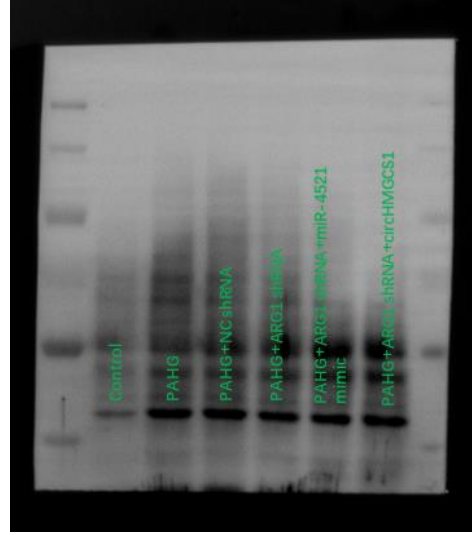

ICAM1

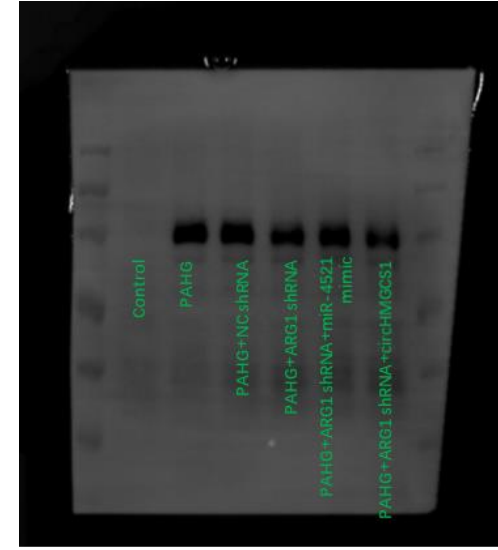

VCAM1

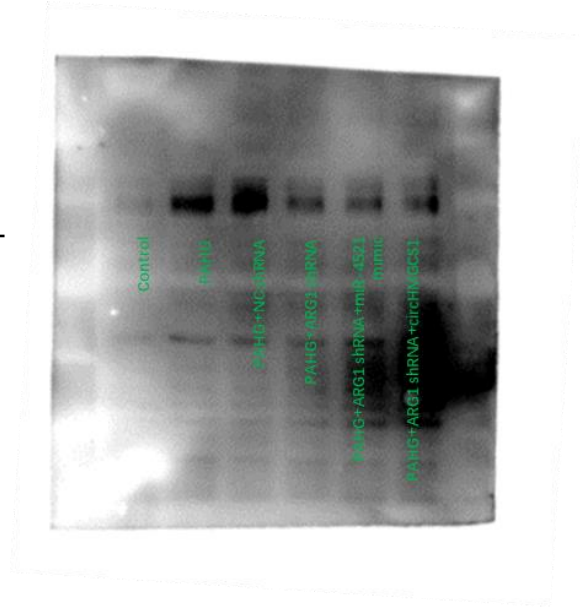

$\beta$ -actin

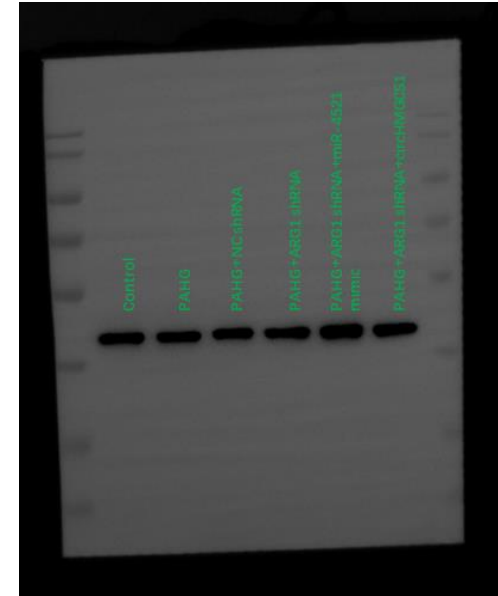

Supplement: Figure 7—source data 1. [file elife-97267-fig7-data1.zip › Figure 7-source data 1/Figure 7-Source data 1 Uncropped and labeled gels for Figure 7.pdf]

ET-1

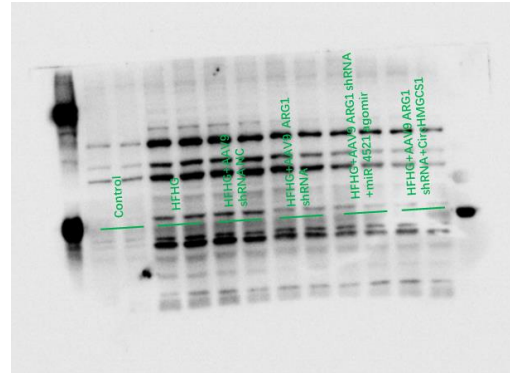

ICAM1

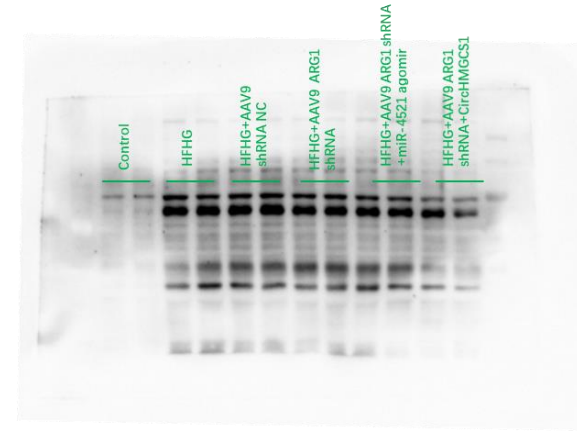

VCAM1

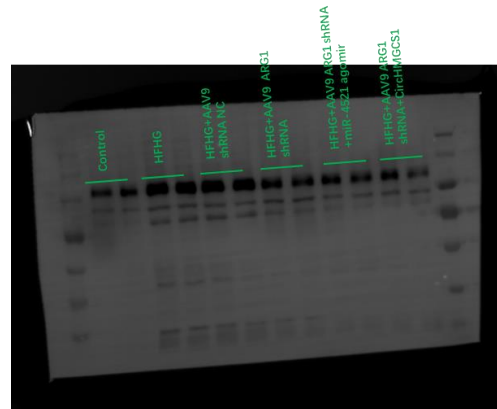

$\beta$ -actin

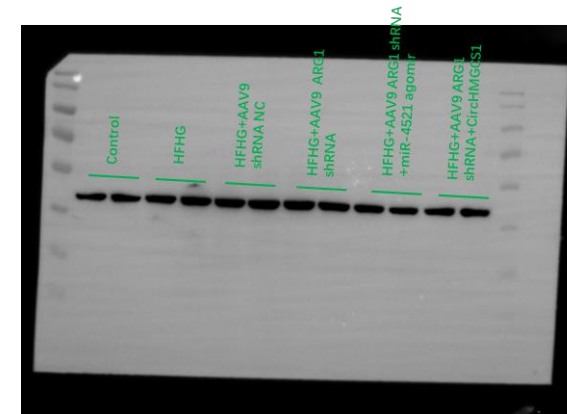

Supplement: Figure 7—source data 1. [file elife-97267-fig7-data1.zip › Figure 7-source data 1/Figure 7-Source data 2 Uncropped and labeled gels for Figure 7.pdf]

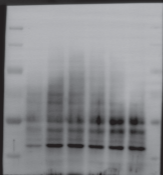

Supplement: Figure 7—source data 2. [file elife-97267-fig7-data2.zip › Figure 7-source data 2/Figure 7-source data 1 Raw unedited gels for Figure 7.pdf]

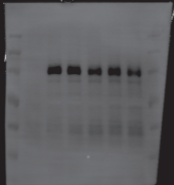

Supplement: Figure 7—source data 2. [file elife-97267-fig7-data2.zip › Figure 7-source data 2/Figure 7-source data 2 Raw unedited gels for Figure 7.pdf]

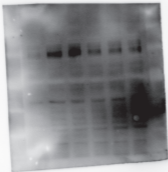

Supplement: Figure 7—source data 2. [file elife-97267-fig7-data2.zip › Figure 7-source data 2/Figure 7-source data 3 Raw unedited gels for Figure 7.pdf]

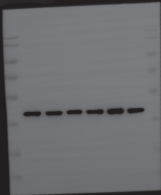

Supplement: Figure 7—source data 2. [file elife-97267-fig7-data2.zip › Figure 7-source data 2/Figure 7-source data 4 Raw unedited gels for Figure 7.pdf]

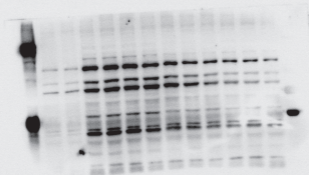

Supplement: Figure 7—source data 2. [file elife-97267-fig7-data2.zip › Figure 7-source data 2/Figure 7-source data 5 Raw unedited gels for Figure 7.pdf]

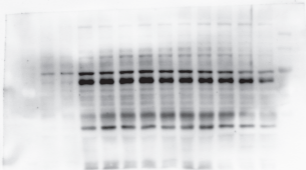

Supplement: Figure 7—source data 2. [file elife-97267-fig7-data2.zip › Figure 7-source data 2/Figure 7-source data 6 Raw unedited gels for Figure 7.pdf]

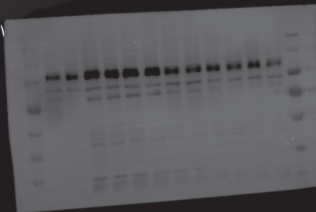

Supplement: Figure 7—source data 2. [file elife-97267-fig7-data2.zip › Figure 7-source data 2/Figure 7-source data 7 Raw unedited gels for Figure 7.pdf]

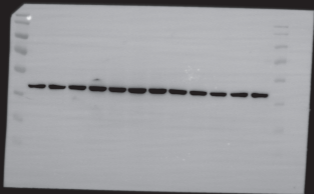

Supplement: Figure 7—source data 2. [file elife-97267-fig7-data2.zip › Figure 7-source data 2/Figure 7-source data 8 Raw unedited gels for Figure 7.pdf]
